# First-in-human study and clinical case reports of the alveolar bone regeneration with the secretome from human mesenchymal stem cells

**DOI:** 10.1186/s13005-016-0101-5

**Published:** 2016-01-15

**Authors:** Wataru Katagiri, Masashi Osugi, Takamasa Kawai, Hideharu Hibi

**Affiliations:** Department of Oral and Maxillofacial Surgery, Nagoya University Graduate School of Medicine, 65 Tsuruma-cho, Showa-ku, Nagoya, 466-8550 Japan

**Keywords:** Secretome, Mesenchymal stem cells (MSC), Tissue engineering, Regenerative medicine, Bone

## Abstract

**Background:**

Secreted growth factors and cytokines in the conditioned medium from bone marrow-derived mesenchymal stem cells (MSC-CM) have several effects on cell behavior. Our previous studies revealed that MSC-CM enhances bone regeneration by increasing cell mobilization, angiogenesis, and osteogenesis in vitro and in vivo. This clinical study was undertaken to evaluate the safety and use of MSC-CM for alveolar bone regeneration in eight patients who were diagnosed as needing bone augmentation prior to dental implant placement.

**Methods:**

The protocol of this clinical study was approved by the ethics committee of Nagoya University Hospital. MSC-CM was prepared from conditioned medium from commercially available human bone marrow-derived MSCs. Patients were treated with beta-tricalcuim phosphate (β-TCP) or an atelocollagen sponge soaked with MSC-CM. Clinical and radiographic assessments were performed during the follow-up period. Histological assessments were also performed in some cases. Clinical and histological data from patients who underwent the SFE procedure without MSC-CM were also used retrospectively as reference controls.

**Results:**

MSC-CM contained several cytokines such as insulin-like growth factor-1, vascular endothelial growth factor, transforming growth factor-β1, and hepatocyte growth factor in relatively low amounts. No systemic or local complications were reported throughout the study. Radiographic evaluation revealed early bone formation in all cases. Histological evaluation also supported the radiographic findings. Furthermore, infiltration of inflammatory cells was scarce throughout the specimens.

**Conclusions:**

MSC-CM was used safely and with less inflammatory signs and appears to have great osteogenic potential for regenerative medicine of bone. This is the first in-human clinical study of alveolar bone regeneration using MSC-CM.

## Background

Alveolar bone regeneration with grafting is often carried out prior to placement of dental implants. Several graft materials have been used including autogenous bone, xenogeneic bone, and synthetic bone substitutes. Autogenous bone grafts have been used for a long time with good predictability and are considered the “gold standard” because of their osteoinductive and osteoconductive properties and immunogenic compatibility. However, autogenous bone must be harvested from a donor site of the patient and is associated with higher morbidity [1, 2]. Xenogeneic bone and synthetic bone substitutes such as deproteinized bovine bone, hydroxyapatite, and calcium triphosphate are often used clinically as osteoconductive scaffolds, but they provide limited osteoinductivity and a potential risk of infection and extrusion [3]. Osteoinductive growth factors such as bone morphogenic protein (BMP)-2 have been used with these osteoconductive materials to promote bone regeneration [4]. However, recent studies have indicated unexpected effects on bone regeneration [5] including induction of a severe inflammatory response, because of the higher dose with clinical application of BMP-2 [6–8].

Recently, the concept of tissue engineering and regenerative medicine has been widely accepted [9], and many clinical studies have been performed including studies of bone and periodontal regenerative medicine.

We previously developed a technique whereby autogenous human mesenchymal stem cells (hMSCs) from the patient’s bone marrow are combined with platelet-rich plasma for use as an alternative to such materials with predictable good prognosis [10, 11]. However, clinical use of stem cells requires highly qualified safety investigation and quality management of cell handling, and is very expensive. These limitations currently impede the widespread use of stem cells for alveolar bone regeneration therapy. Moreover, recent studies have revealed that the implanted cells do not survive long [12–14]. As an alternative, the effects of the secretomes, the various factors secreted into the medium, from stem cells on tissue repair and regeneration have attracted much attention [15–17].

We have reported the effects of the secretomes in the conditioned medium from bone marrow-derived mesenchymal stem cells (MSC-CM) on bone and periodontal tissue regeneration in vitro and in vivo. MSC-CM contains several cytokines such as insulin-like growth factor (IGF)-1, vascular endothelial growth factor (VEGF), and transforming growth factor (TGF)-β1. MSC-CM enhances cell proliferation, mobilization, angiogenesis, and expression of osteogenic markers such as *alkaline phosphatase*, *collagen type I*, and *Runx2* genes [18]. MSC-CM also recruits endogenous stem cells to the grafted site and shows early bone and periodontal regeneration in rat calvarial bone defects and dog periodontal bone defects [19–21]. Furthermore, the concentrations of cytokines contained in MSC-CM are relatively low such that use of MSC-CM does not induce the severe histological inflammatory responses that are observed with the clinical use of recombinant human BMP-2 [18].

Based on these experimental and preclinical studies, we performed a clinical study using MSC-CM for alveolar bone regeneration. Until now, no human study has been reported, and thus, this is the first in-human study using MSC-CM for bone regenerative therapy.

## Methods

### Cell culture and preparation of MSC-CM

Human MSCs were purchased from Lonza Inc. (Walkersville, MD, USA) and cultured in mesenchymal stem cell basal medium (MSCBM; Lonza Inc.) with MSCGM SingleQuots (Lonza Inc.). Cells of the third passage were used in this study. Cells were maintained at 37 °C in 5 % CO_2_/95 % air.

When hMSCs reached 70 % confluency, the medium was refreshed with 10 ml serum-free Dulbecco’s modified Eagle’s Medium (DMEM; GIBCO, Rockville, MD, USA) containing antibiotics (100 units/ml penicillin G, 100 μg/ml streptomycin, and 0.25 μg/ml amphotericin B; GIBCO). The cell culture-conditioned media were collected after incubation for 48 h. This medium was defined as MSC-CM.

For clinical use, MSC-CM was then concentrated and stored as described below. Briefly, MSC-CM was centrifuged for 5 min at 1500 rpm and then for another 1 min at 15,000 rpm to remove any cells. Five milliliters MSC-CM was mixed with 45 ml 100 % ethanol and incubated at −20 °C for 1 h. The mixture was centrifuged for 15 min at 15,000 rpm at 4 °C, and the supernatant was discarded. The precipitate was suspended again in cold 90 % ethanol and centrifuged for 15 min at 15,000 rpm at 4 °C. The final precipitate was frozen at −80 °C, lyophilized, and stored at −80 °C until use.

To verify the safety, MSC-CM was examined for not only contamination with bacteria, fungi, or mycoplasmas but also virus infection including hepatitis B and C virus, human immunodeficiency virus, and human T-cell leukemia virus before the following procedures.

### Enzyme-linked immunosorbent assay (ELISA)

The levels of IGF-1, VEGF, TGF-β1, hepatocyte growth factor (HGF), fibroblast growth factor (FGF)-2, platelet-derived growth factor (PDGF)-BB, BMP-2, and stromal cell-derived factor (SDF)-1α in MSC-CM were investigated using ELISA. The concentration of these factors was measured using a Human Quantikine ELISA kit (R&D Systems, Minneapolis, MN, USA) according to the manufacturer’s instructions. Briefly, 200 μl MSC-CM, DMEM-0 % FBS, or DMEM-30 % FBS were added to 96-well microplates that were coated with a monoclonal antibody recognizing the factor of interest and incubated for 2 h. After washing with phosphate-buffered saline (PBS), a horseradish peroxidase-conjugated antibody against the cytokine or growth factor was added to each well, incubated for 2 h, and washed. Substrate solution was added and incubated for 30 min, and the reaction was terminated by addition of the stop solution. Cytokine levels were determined by measuring the optical density at 450 nm using a microplate spectrophotometer (Benchmark Plus; Bio-Rad, Hercules, CA, USA).

### Patient selection

Eight partially edentulous patients who were diagnosed as needing bone augmentation, including maxillary sinus floor elevation (SFE), guided bone regeneration (GBR) and socket preservation (SP), were enrolled in this study. Because of severe alveolar bone atrophy, all the patients had problems with retention of conventional removable dentures, and these procedures and application of dental implants were expected to solve this problem. The criteria for application of these procedures are <5 mm residual bone from the sinus floor to the alveolar ridge in SFE cases and <10 mm of residual bone height in GBR cases.

After routine oral and physical examinations, patients who did not desire to undergo surgery for autogenous bone harvesting were selected for this study. All patients were healthy and free from any disease that may have influenced the outcome of this study (e.g., diabetes, malignant tumor, autoimmune disease, bone disease, endocrine disease). Each patient provided informed consent after receiving detailed information about the surgical procedures, graft materials, alternative treatments, and uncertainties of using a new method. The ethics committee of Nagoya University Hospital approved this research protocol (No.3437). Clinical, radiographic and histological data from two patients who underwent the SFE procedure without MSC-CM were also used retrospectively as reference controls.

### Preparation and application of MSC-CM

**Fig. 1:**
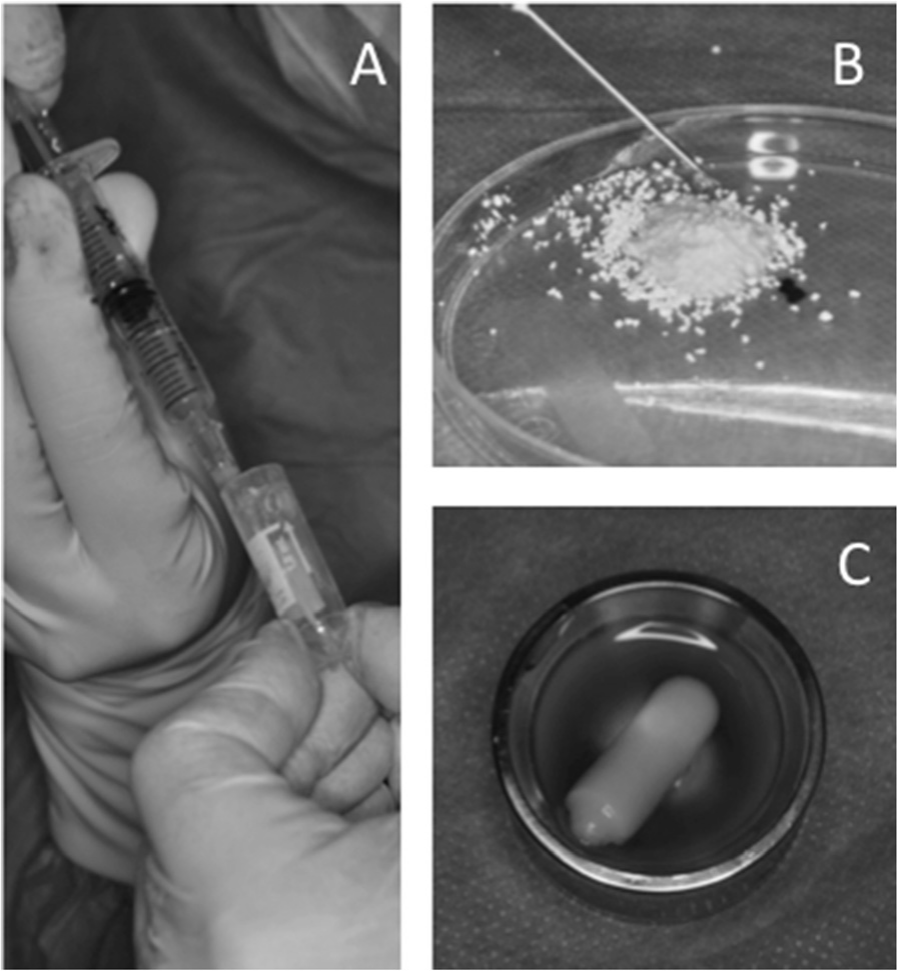
Preparation of MSC-CM for implantation with different scaffolds. **a** Lyophilized MSC-CM is dissolved in saline during the surgery. **b** MSC-CM is mixed with β-TCP. **c** An atelocollagen sponge is soaked in MSC-CM solution

During the surgery, MSC-CM was dissolved in 5 ml saline. As a scaffold, porous pure beta-tricalcuim phosphate (β-TCP; Osferion®, Olympus Terumo Biomaterials, Tokyo, Japan) was used in SFE and GBR cases. In some cases with small and four-wall defects in SP cases, a shell-shaped atelocollagen sponge (ACS) (Terudermis®; Olympus Terumo Biomaterials) was also used as a scaffold. β-TCP (1 g) or ACS (8 mm in diameter × 25 mm in length) was soaked with 3 ml of MSC-CM solution for at least 5 min and then used for grafting (Fig. 1).

### Surgical procedures

All surgical procedures were conducted under local anesthesia. The SFE procedure was performed using the lateral window approach [22]. Briefly, a window was created with a round diamond burr at the lateral maxillary sinus wall. After removing the bone, the sinus membrane was elevated. The space created by this procedure was filled with the mixture of MSC-CM and β-TCP (MSC-CM/β-TCP). In cases of simultaneous implant placement, Brånemark MkIII Groovy or NobelActive implants (Nobel Biocare, Zürich, Switwerland) were placed into the alveolar bone at a depth of at least 5 mm according to pre-surgical computer simulation. The lateral window was then covered with a bioabsorbable poly-lactic acid-glycol acid (PLGA) membrane (GC Membrane®, GC, Tokyo, Japan). The mucoperiosteal flap was repositioned and sutured in the usual manner. The GBR procedure was performed after the mucoperiosteal flap was elevated. In cases of simultaneous implant placement, implants were placed in the same manner as described above. Because of the atrophic alveolar bone, exposed threads of the implants were covered with MSC-CM/β-TCP. In SP cases such as tooth extraction sockets, the bone defects were filled with MSC-CM/ACS. After grafting MSC-CM/β-TCP, the grafted areas were covered with PLGA membranes. Lesions grafted with MSC-CM/ACS were not covered with any membranes, and all lesions were closed with a tension-free mucoperiosteal flap. In all cases, the second-stage surgeries were performed about 6 months after implant placement (first-stage surgery). All surgeries were performed by the same surgeon (W.K.).

### Clinical and radiographic observations

After registration, safety evaluations were performed before surgery. Briefly, the drug lymphocyte stimulation test (DLST) and the patch test were performed to evaluate the allergic reaction to MSC-CM. Blood tests were also performed to check for any organ dysfunction and inflammatory and allergic reactions before and after surgery. Computed tomography (CT) scans and panoramic X-ray examinations were performed before and after surgery. Bone biopsies were obtained for histology with a 2-mm-diameter trephine bar at the regenerated bone areas during the second-stage surgery and immersed in 10 % formaldehyde. Paraffin sections were prepared according to standard protocols, and hematoxylin-eosin staining was performed. The protocol for clinical and radiographic observations in this study is shown in Table 1.

**Table 1:**
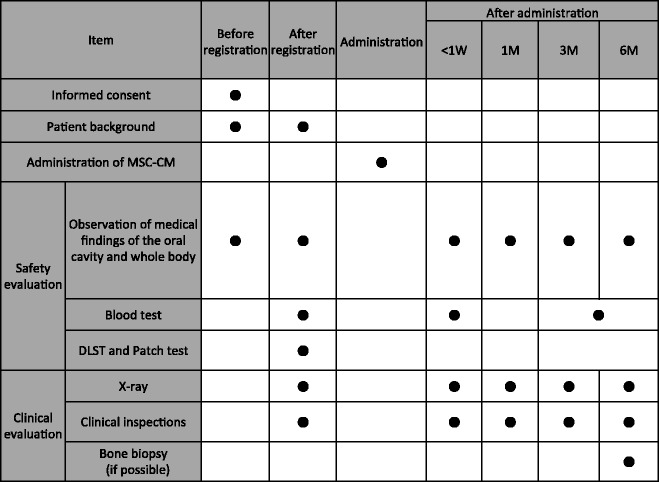
The schedule of this clinical study

## Results

### Cytokines present in MSC-CM

The concentrations of the cytokines IGF-1, VEGF, TGF-β1, HGF, FGF-2, PDGF-BB, BMP-2, and SDF-1α in MSC-CM were quantified with ELISA. Cytokines were not detected in DMEM-0 % and DMEM-30 %. However, MSC-CM contained IGF-1, VEGF, TGF-β1, and HGF at concentrations of 1386 ± 465, 468.5 ± 109, 339.8 ± 14.4, and 20.3 ± 7.8 pg/ml, respectively. The other factors assayed were not detected in MSC-CM (Table 2).

**Table 2:** Cytokines detected in MSC-CM

Cytokines	Concentration (pg/ml)
IGF-1	1386±465
VEGF	465.8±109
TGF-β	339.8±14.4
HGF	20.3±7.8
FGF-2	ND
PDGF-BB	ND
BMP-2	ND
SDF-1	ND

*ND* Not detected

### Clinical observations

The eight patients in this study included three men and five women, ranging from 45 to 67 (mean, 57.8 years) years old. SFE was performed in three patients, and GBR was performed in five patients. β-TCP was used in all SFE cases and two GBR cases, whereas ACS was used in two SP cases (Table 3). No systemic or local complications were noted throughout the study. The results of DLST and blood tests showed no abnormal findings except minor inflammatory signs after surgery. Implant placement was performed simultaneously with the bone augmentation procedure in five cases and was performed within 8 to 9 months after the first surgeries in two cases. All the implants were placed without any problems and showed good initial stabilities.

**Table 3:** Patient data

Patient	Age(y)	Sex	Location	Surgery	Scaffold
1	46	F	25,26	SFE	TCP
2	67	F	45	SP	ACS
3	45	F	35,36	GBR	TCP
4	51	F	47	SP	ACS
5	63	M	11,21	GBR	TCP
6	67	F	45	SP	ACS
7	59	M	27	SFE	TCP
8	64	M	25,26,27	SFE	TCP

*SFE* sinus floor elevation, *GBR* guided bone regeneration, *SP* socket preservation, *TCP* β-TCP, *ACS* atelocollagen sponge

### Radiographic observations

Panorama X-ray and CT images showed early mineralization in the augmented bone. Furthermore, CT images showed that the β-TCP structures gradually become indistinct around 6 months after the first surgery. No bone resorption was observed in any cases, and notable edematous swelling of the maxillary sinus membrane was not obvious throughout the study after the surgery in SFE cases.

### Histological observations

Bone biopsies were taken from five cases. Newly formed bone was observed in each specimen. Resorption of β-TCP was found, and replacement with new bone had occurred only 6 months after the first surgery. Furthermore, infiltration of inflammatory cells (e.g., neutrophils, macrophages) was observed less often in these specimens.

## Case reports

### Case 1

**Fig. 2:**
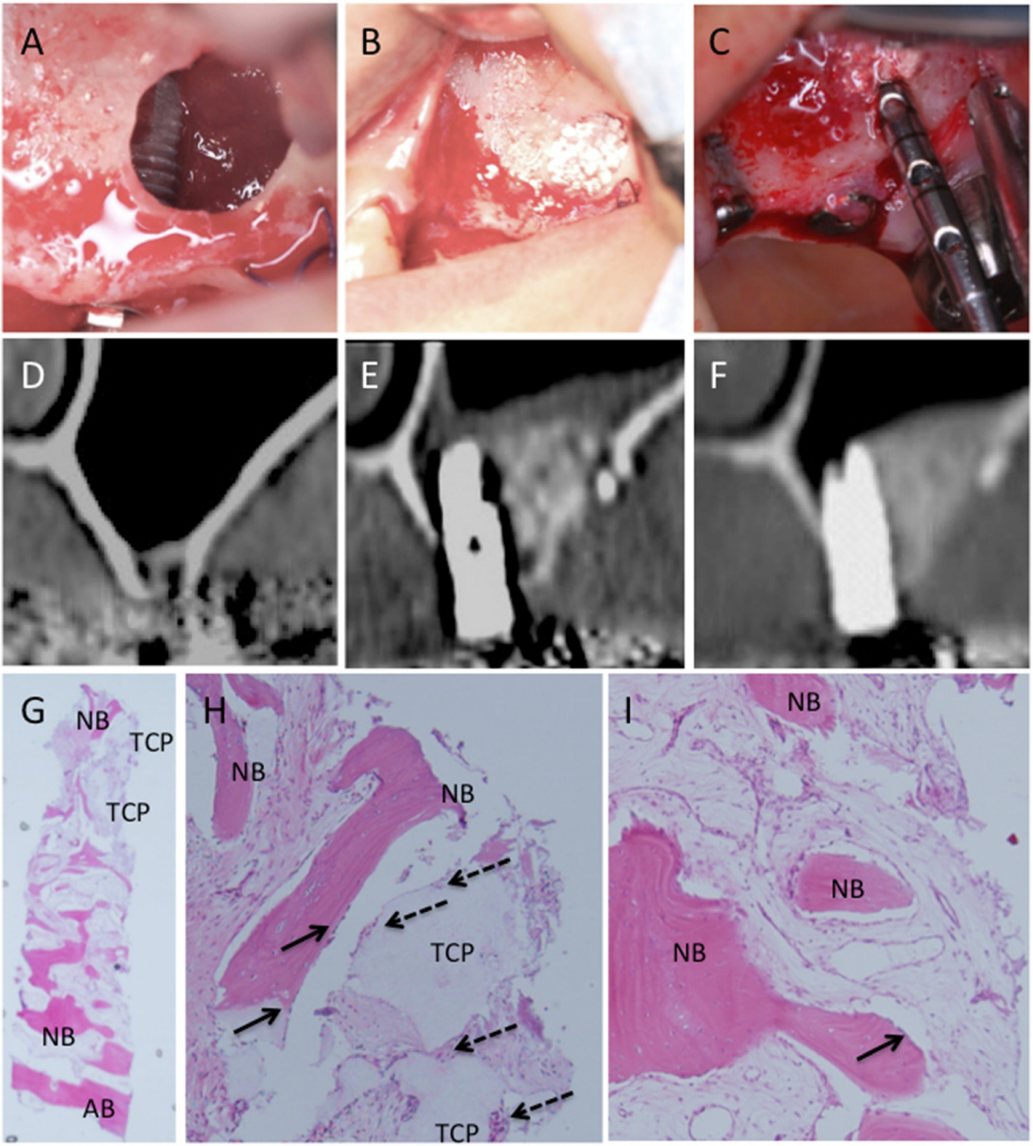
Clinical, radiographic, and histological observations in Case 1. **a** The implant is placed into the cavity created by the SFE procedure. **b** The cavity is filled with MSC-CM/β-TCP. **c** The second surgery. A bone biopsy was taken from the newly formed bone 6 months after the SFE procedure. **d**-**f** CT images before and after the SFE procedure. **d** Before the SFE procedure. **e** Three months after the SFE procedure. **f** Six months after the SFE procedure. **g**, **h**, **i** Histologic findings of the specimen from the augmented area (H-E stain). **g** Out line of the specimen (×12.5). **h** Nasal side of the specimen (H; ×100). **i** Oral side of the specimen (×100). Newly formed bone was seen throughout the specimen. Arrow indicates the arrangement of osteoblasts along with the newly formed bone. The residual β-TCP granules were seen at the nasal side of specimen. The dotted arrow indicates β-TCP resorption by osteoclasts. (TCP: β-TCP, NB: Newly formed bone)

A 46-year-old woman came to the hospital because she had lost her left maxillary teeth because of periodontitis about 3 months before. CT images showed alveolar bone loss at 25 and 26; the thickness of the residual bone was about 3 to 5 mm. SFE and simultaneous implant placement were planned under local anesthesia. Once the lateral maxillary window was opened and the maxillary sinus membrane was elevated, two Brånemark MkIII Groovy 11.5-mm long implants were placed into the prepared cavity (Fig. 2a). Then MSC-CM/β-TCP was implanted into the cavity, especially around the exposed implants. About 0.5 g β-TCP was used to fill the cavity (Fig. 2b). The window was covered with a PLGA membrane, and the wound was closed with a tension-free mucoperiosteal flap. After 6 months, the second-stage surgery was performed. The lateral window was almost covered with newly formed bone and some remnants of β-TCP. Osseointegration of the implants was good, and a bone biopsy was taken from the augmented area (Fig. 2c).

**Fig. 3:**
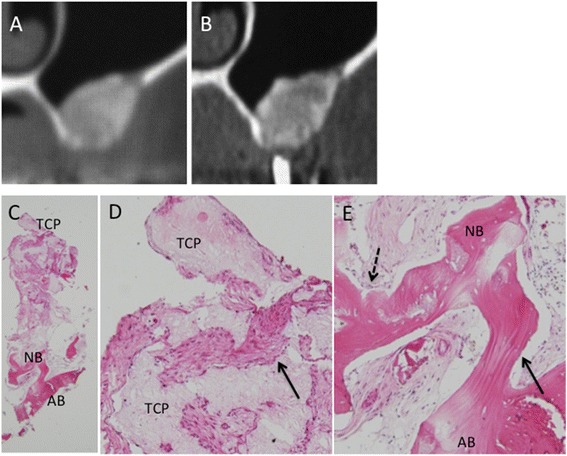
Histological observation in the reference case. CT images and histology of an SFE case with β-TCP alone are shown. **a**, **b** CT images after the SFE procedure. **a** Three months after the SFE procedure. **b** Six months after the SFE procedure. **c** Outline of the specimen. Newly formed bone is scarce, and β-TCP remains much from nasal part to the middle area of the specimen (H-E, ×12.5). **d** Nasal side of the specimen (×100). **e** Oral side of the specimen (×100). Replacement of new bone is insufficient. The arrow indicates infiltration of inflammatory cells. Arrow indicates the arrangement of osteoblasts along with the newly formed bone. The dotted arrow indicates that osteoclasts, but the number of osteoclasts was less. Infiltration of the inflammatory cells was seen in the oral part of the specimen. (TCP: β-TCP, NB: Newly formed bone, AB: Alveolar bone)

In CT images, the particles of implanted β-TCP were clear 1 month after the first surgery, but became gradually indistinct after 6 months (Fig. 2d-f). Histologic findings also supported this phenomenon. The remnants of β-TCP were resolved from the edge, and replacement with new bone was observed. Interestingly, infiltration of inflammatory cells was not severe, and newly formed bone was seen throughout the specimen (Fig. 2g, h). As a reference control, histology from a patient who had undergone SFE with β-TCP only (i.e., without MSC-CM) 6 months before is shown in Fig. 3. Replacements of β-TCP with regenerated bone were scarce, and infiltration of inflammatory cells was observed around the particles of β-TCP.

### Case 2

**Fig. 4:**
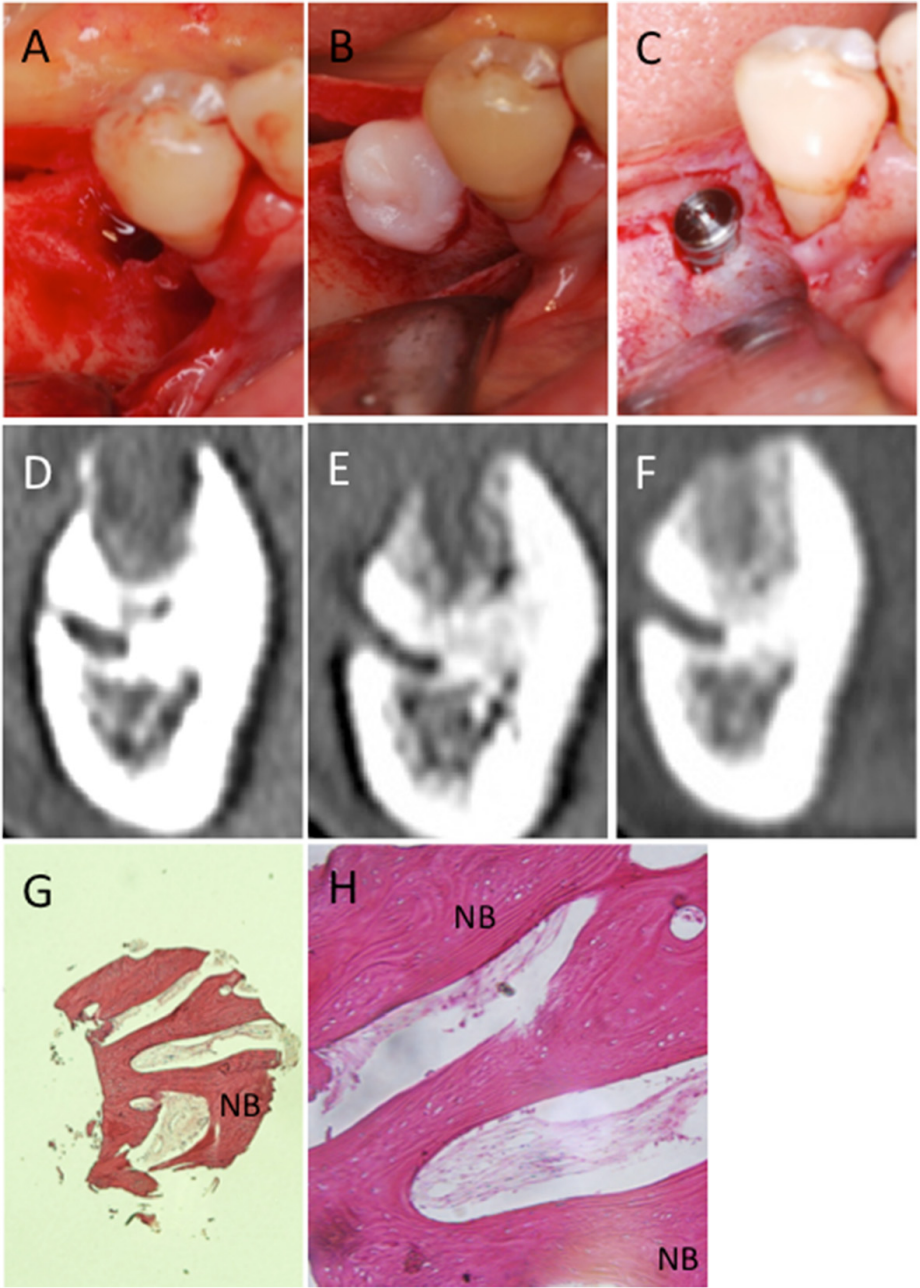
Clinical, radiographic, and histological observations in Case 2. **a** The extraction socket remains at the distal part of the right mandibular first premolar. **b** The socket was filled with MSC-CM/ACS. **c** The implant placement 8 months after MSC/ACS implantation. The socket is completely regenerated with newly formed bone. A bone biopsy was done where the implant was placed. **d**-**f** CT images before and after the SP procedure. **d** Before the SP procedure. **e** Three months after the SP procedure. **f** Six months after the SP procedure. **g**, **h** Histologic findings of the specimen from the augmented area (H-E stain) (G; ×12.5 and H; ×40). Newly formed and mature bone was seen throughout the specimen. No inflammatory cells were seen in this specimen. (NB: Newly formed bone)

A 67-year-old woman presented with severe bone resorption due to periodontitis at 45, and this tooth was extracted 3 weeks prior to the SP procedure. X-ray diagnosis revealed that the residual bone height was appropriate. However, the extraction socket remained, and socket preservation was considered to be ideal for future implant placement. A crestal incision and mucoperiosteal flap were made at the buccal aspect of the right mandibular molar area. Granulation tissue was removed from the flap and bony defect with curettes, and perforation of the surrounding cortical bone was done with a bar to improve the blood supply. MSC-CM/ACS was implanted into the bony defect, and the flap was closed (Fig. 4a, b). Healing was uneventful. CT images showed gradual bone formation in the implanted area (Fig. 4d-f). Six months after MSC-CM/ACS implantation, CT images showed that the extraction socket had almost completely filled with newly formed bone, and placement of the implant was planned 8 months after the first surgery. Prior to implant placement, a bone biopsy was done from where the NobelActive 11.5-mm-long implant would be placed (Fig. 4c). The bone quality was so good that the implant acquired initial stability. H-E staining of the specimen showed dense trabecular bone with little infiltration of inflammatory cells (Fig. 4g, h).

## Discussion and conclusions

This study evaluated the safety and efficacy of MSC-CM in a human clinical trial for alveolar bone regeneration. Tissue engineering and regenerative medicine of the bone and periodontal tissue using stem cells has begun to be used clinically [10, 23]. We have developed regenerative medicine for bone and periodontal tissue using autogenous bone marrow-derived MSCs, and this approach is considered an alternative to conventional autogenous bone grafting [11]. However, the use of adult stem cells for tissue regeneration has several problems such as safety and quality management in stem cell handling, and the high cost and strict regulation by authorities currently prevent the popularization of this approach. Moreover, permanent engraftment and transdifferentiation of transplanted adult stem cells have not been confirmed [24]. Recent studies have indicated that the therapeutic effects of transplanted stem cells are considered effective for tissue regeneration and for their interesting role as cellular modulators, not just for their multipotent differentiation ability [25, 26].

Transplanted stem cells release several paracrine factors such as cytokines, growth factors, and extracellular matrix molecules that modulate endogenous cellular mobilization, angiogenesis, and cellular differentiation, and induce endogenous stem cells to promote tissue repair and regeneration [15]. Our previous studies revealed that MSC-CM contains several cytokines, such as IGF-1, VEGF, TGF-β1, and HGF, and enhances early bone and periodontal tissue regeneration [18–21]. IGF-1 induces osteoblast proliferation and migration [27, 28] and enhances periodontal regeneration by stimulating periodontal ligament (PDL) cells through the PI3K pathway [29]. VEGF is believed to be the main regulator of angiogenesis, and bone marrow stromal cells secrete sufficient quantities of VEGF to enhance the survival and differentiation of endothelial cells. VEGF also contributes to osteogenesis [30]. TGF-β1 enhances the migration of osteoprogenitor cells, and regulates cell proliferation, differentiation, and extracellular matrix production [31]. TGF-β1 also stimulates PDL regeneration and repair [32] and is expressed during the development of the alveolar bone, PDL, and cementum [33]. HGF has a direct effect on angiogenesis [34]. A previous study demonstrated that a combination of several factors has an additive effect on cellular migration and osteogenic differentiation [35]. Some studies have investigated a combination of two or more factors to promote bone regeneration [35, 36].

In our study, the advantage of a combination of several factors seems to be versatile effects on bone regeneration. Furthermore, the concentration of each cytokine in MSC-CM may be relatively low and at physiological levels because they were secreted from cells and are not an industrial product such as recombinant BMP-2. BMP-2 has a strong osteoinductive ability, but a high concentration of BMP-2 is required to obtain therapeutic effects on bone regeneration. Cowan et al. suggested that BMP has dose-dependent bone regeneration effects in the rat calvaria model, and the concentration of BMP-2 that induced effective bone regeneration was 240 ng/mm^3^ [37]. In MSC-CM, the concentration of each cytokine was around hundreds to thousands of picograms per milliliter.

MSC-CM has other effects as well. Ionescu et al. suggested that conditioned medium from MSCs promotes alternative macrophage activation, producing a wound healing/anti-inflammatory M2 phenotype that is due in part to IGF-1 [38]. We found that only a few inflammatory cells were seen where MSC-CM was implanted in our allogeneic animal study [18–21]. In this clinical study, no patient showed abnormal swelling or delayed healing after surgery. β-TCP has been widely used clinically both in orthopedic and maxillofacial lesions and produces excellent osteoconductivity; however, β-TCP is resorbed over a long period [39]. In this study, β-TCP mixed with MSC-CM promoted early resorption and replacement of new bone compared with β-TCP without MSC-CM. This phenomenon may have been due not only to the effects of several cytokines that induced several types of cell behavior and angiogenesis as described above, but also alternative macrophage phenotypes that quickly degraded the scaffold. Badylak et al. investigated the two different types of scaffolds. One was a chemically cross-linked scaffold that did not show any significant degradation during the study period following implantation and that resulted in a predominantly M1-type macrophage response. The other was a non-cross-linked scaffold that was rapidly degraded following implantation and that elicited an M2-type response and showed more constructive regeneration of tissue [40]. From this point of view, the cytokines in MSC-CM are considered to induce the resorption of β-TCP and the switching of the phenotype of macrophages from M1 to M2 at an earlier phase of bone regeneration. Interestingly, implantation of MSC-CM/ACS resulted in denser new bone formation shown as in case 2. ACS is resorbed more easily than β-TCP, and this finding is also consistent with the relationship between the mechanical characteristics of the scaffolds and the phenotype of macrophages.

MSC-CM leads to prominent osteoinductivity according to our series of studies. MSC-CM is required at a relatively low physiological dose to produce therapeutic effects, and it may also have an immunomodulatory effect as described above. Further studies will be required to confirm which individual factors are important and indispensable. This implies the possibility of making new agents using these novel concepts for not only bone and periodontal regeneration but also other systemic diseases that had previously been expected to be treated with transplantation of stem cells.

This is the first-in-human study for alveolar bone regeneration using MSC-CM. Because this study was aimed to assess the safety and response to MSC-CM clinically with a small patients group, we could not deeply show and discuss about the radiographic and histological effects. We have already started the next phase clinical trial to assess the efficacy of MSC-CM on alveolar bone regeneration with more lager patients group. We are planning to report these results will in the future.

## Abbreviations

MSC-CM: conditioned medium from bone marrow-derived mesenchymal stem cells
β-TCP: beta-tricalcium phosphate
ACS: atelocollagen sponge
IGF: insulin like growth factor
VEGF: vascular endothelial growth factor
TGF: transforming growth factor
BMP: bone morphogenetic protein
HGF: hepatocyte growth factor
FGF: fibroblast growth factor
PDGF: platelet-derived growth factor
SDF: stromal cell-derived factor
DMEM: Dulbecco’s modified eagle’s medium
SFE: sinus floor elevation
GBR: guided bone regeneration
SP: socket preservation
